# Current Indications for Intraarterial Chemotherapy in Neurointerventional Surgery

**DOI:** 10.1161/SVIN.122.000425

**Published:** 2022-11-07

**Authors:** Bryan T. Mott, Ankitha Iyer, Eleanor Smith, Kyle Fargen, Patrick Brown, Stacey Quintero Wolfe

**Affiliations:** ^1^ Department of Neurological Surgery Wake Forest School of Medicine Winston‐Salem NC

**Keywords:** CNS lymphoma, glioblastoma multiforme, head and neck squamous cell cancer, intraarterial chemotherapy, neuroendovascular, neurointerventional, retinoblastoma

## Abstract

**Background:**

In pursuit of a cure for cancer, it is imperative to utilize every tool, resource, and strategy. Included under this umbrella is the optimization of drug delivery. Broadly speaking, anti‐cancer treatment has been perpetually hindered by off‐target activity, systemic toxicity, and other adverse reactions. Intraarterial (IA) delivery of therapeutics is an approach that has garnered increased attention in recent years. This approach can deliver drug directly to the desired site with the potential to minimize systemic toxicity.

**Methods:**

In this review, we briefly cover existing IA indications for peripheral solid tumors as a base from which we can learn, followed by trials and procedural considerations of IA drug delivery for neck, head, and central nervous system tumors.

**Results:**

While the bulk of IA research and clinical trials have focused on drug delivery outside of the central nervous system, there have been recent encouraging results in IA tumor treatment within the neurointerventional arena, such as head and neck tumors, retinoblastoma, glioblastoma multiforme, and central nervous system lymphoma.

**Conclusion:**

This review highlights the need for increased clinical research on IA chemotherapeutic delivery as a multi‐disciplinary approach involving neurointerventional surgeons.

**Nonstandard Abbreviations and Acronyms**

BBB: blood–brain barrier
GBM: glioblastoma multiforme
IA: intraarterial
IV: intravenous
PCNSL: primary central nervous system lymphoma

**Clinical Perspective**

- Intraarterial chemotherapy is standard of care in several solid body tumors, and has shown signals of efficacy in many neurointerventional areas such as glioblastoma multiforme, central nervous sytem lymphoma, head and neck tumors, and retinoblastoma.
- Intraarterial chemotherapy should be explored in well‐designed future clinical trials, and neurointerventionalists should be closely involved with planning these trials.
- Intraarterial chemotherapy should be considered for treatment of advanced retinoblastoma, with a 75% eye salvage rate.

Cancer remains an insidious disease, often without cure, however, significant strides for less‐invasive treatment options are on the horizon to reduce the morbidity of surgery, improve disease control and increase survival. With the advent of biologic therapies such as immunotherapy and small molecule kinase inhibitors, the landscape of cancer treatment is changing, and the route of delivery may necessitate change, as well. Intraarterial (IA) delivery of chemotherapeutic agents has garnered renewed interest based on improved delivery devices and novel therapeutics. Currently, drug administration is dominated by intravenous (IV) delivery because it is straightforward and facilitates excellent volume of distribution. However, it is a systemic rather than localized therapy with consequent toxicities and potential need for larger doses to combat metabolism. For these reasons, IA drug administration may be advantageous, particularly in delivery of chemotherapeutics directly into the tumor bed. By delivering drug locally, it may be possible to optimize the overall dose of drug required, maximize the effectiveness of the dose given by limiting first pass metabolism, reduce off‐target drug activities and other unwanted or dose‐limiting toxicities, and improve patient compliance due to improved treatment profile. This may be particularly appealing for established and emerging immunotherapeutics, in that a local immune response can be stimulated while simultaneously limiting systemic inflammatory reactions. In this review, we outline the existing state of IA delivery of chemotherapy with a particular focus on its application for neoplastic disease of the neck, head, and brain, and discuss future areas of inquiry and growth for this technique.

## Pharmacokinetic Considerations

IA delivery of chemotherapy carries several considerations. First, conversion from IV to IA delivery may alter the pharmacokinetic profile of the drug.^1^ For most chemotherapeutics, such as cisplatin, the dose, concentration, volume of distribution, rate, and frequency of administration are well established to maximize antitumoral efficacy while limiting side effects such as nephrotoxicity.^2^ When a drug is given intraarterially, each of these parameters must be independently established. Can more drug be given because systemic exposure is in fact lower? This appears to be the case in the RADPLAT protocol for squamous cell carcinoma of the head and neck described below (IA Chemotherapy for the Treatment of Head and Neck Tumors), in which the standard cisplatin dose of 75–100 mg/m^2^ was increased to 150 mg/m^2^. Alternatively, it may be that less drug can be given overall due to increased efficacy from higher local concentration, as seen with methotrexate for treatment of CNS lymphoma (see IA Chemotherapy for Glioblastoma and Other CNS Tumors). Other parameters such as rate and frequency of administration will hinge on clinical response of the tumor and the side effect profile, and clinical evaluation will be necessary to establish them.

A primary focus of pharmacokinetics is to maximize drug uptake into tumors. This is especially important in CNS‐based tumors because the drug must pass through the blood–brain barrier (BBB). The BBB is comprised of a unique arrangement of endothelial cells that have continuous intercellular tight junctions. Under normal circumstances, molecular transport across these membranes is primarily conducted by specific transporters.^3^ When considering the physical process of molecular transport across these membranes in the setting of dynamic blood flow, numerous strategies are being evaluated to assure that drug is taken into the tumor and not simply dispersed into surrounding blood vessels which will result only in systemic distribution that mirrors IV administration. Super‐selective IA cerebral chemotherapeutic infusion may require adjunct techniques,^4^ such as 1) pre‐infusion IA administration of hyperosmolar agents like mannitol or use of etopside to improve local BBB permeability;^5^ 2) external energy sources such as laser interstitial thermal therapy,^6^ magnetic‐resonance guided focused ultrasound (MRgFUS),^7^, ^8^, ^9^ or irreversible electroporation^10^ to physically drive apart the tight junctions of the BBB; and/or 3) flow arrest to increase dwell time and prolong diffusion of drug into the tumor.^11^, ^12^, ^13^ These techniques capitalize on physical phenomena to circumvent the tight junctions of the BBB which function to facilitate specific transport system across the endothelial cells. Currently, IA mannitol administration is most commonly used to increase BBB permeability for current CNS IA applications with the most common regimens being 25% IA mannitol, 3 to 10 mL/sec over 30 seconds, or 20% IA mannitol, 12.5 mL over 120 seconds. Through use of these and other methods, IA delivery becomes a viable treatment option for multiple types of cancer.

## Current Indications of IA Chemotherapy for Solid Tumors

IA chemotherapy was first used over 70 years ago. In 1950, IA delivery of nitrogen mustard was used to treat lymphoma.^14^ Reports of IA therapy for melanoma,^15^, ^16^ hepatocellular carcinoma,^17^, ^18^, ^19^, ^20^, ^21^, ^22^, ^23^, ^24^ pancreatic cancer,^25^, ^26^, ^27^ lung cancer,^28^ bladder cancer,^29^ gastric cancers,^30^ uterine and cervical cancer,^31^, ^32^ prostate cancer,^33^ colorectal cancer, osteosarcoma^34^ and breast cancer^35^ have been published since then with varying degrees of success. Currently, there are over 60 active clinical trials evaluating IA chemotherapy for most solid tumor types (Supplemental Material Table S1), which underscores the enthusiasm for this approach. A full review of IA chemotherapy for solid tumors is beyond the scope of this paper, however, it is worth highlighting at least one example of successful IA therapy which has become standard practice to provide lessons for IA chemotherapy use in the neurointerventional space. Hepatocellular carcinoma is now commonly treated with IA chemotherapy, largely due to endovascular accessibility of the tumor through the hepatic artery and inherent morbidities associated with surgical resection.^36^ There are multiple methods of IA chemotherapy delivery in hepatocellular carcinoma, such as transarterial chemotherapy followed by embolization.^37^ A recent meta‐analysis of 71 non‐randomized and 21 randomized clinical trials involving a total of 6695 patients using different IA approaches and modifications demonstrated clear superiority of IA chemotherapy over systemic chemotherapy alone.^38^ IA chemotherapy for hepatocellular carcinoma highlights the efficacy of IA drug delivery, the versatility of IA chemotherapy, and its potential to be a first‐line treatment option.

## IA Chemotherapy for the Treatment of Head and Neck Tumors

Head and neck cancers (HNCs) possess several unique features affecting clinical management. The majority of HNCs are diagnosed at an advanced stage, often involving regional lymph nodes (stage III or IV.).^39^ Because prognosis correlates strongly with stage at diagnosis, 5‐year overall survival often does not exceed 60% even with aggressive management.^40^, ^41^, ^42^ Surgical approaches to advanced HNC often result in significant functional and cosmetic disability secondary to necessary resection of the larynx, pharynx, and tongue. Squamous cell carcinoma (SCC) represents around 90% of HNCs with the standard of care being chemoradiation.^43^, ^44^ Cisplatin is the central agent of HNC chemotherapy based on dose‐dependent tumor response rates.^45^ Acquired tumor resistance to cisplatin can occur rapidly (after as few as 2–4 cycles)^46^ and may be overcome by increasing the dose, but systemic toxicities of the drug, most notably nephrotoxicity, are a major limiting factor. Radiation‐induced mucositis is also more prevalent with chemoradiation. These treatment‐associated morbidities frequently result in treatment delays and dose reductions, adversely impacting tumor control and overall survival.

As the field of neurointervention has expanded, neurointerventionalists commonly work through the external carotid artery in the treatment of dural arteriovenous fistulae, trauma, and epistaxis, to name a few. This capability brings the potential to treat HNC through IA means into the neurointerventional purvue. IA chemotherapy for HNC was developed to maximize the benefits of chemoradiation and limit dose‐related systemic toxicities. First described by Robbins et al. in the 1990s,^47^, ^48^ the RADPLAT protocol involved an IA cisplatin infusion into the dominant artery or arteries supplying the tumor at a “decadose” of 150 mg/m^2^ over 3 to 5 minutes, directly into the tumor bed. The infusion could be split between 2 and 3 dominant arterial branches. Simultaneous IV infusion of the chelating agent sodium thiosulphate protects vital and peripheral organs by neutralizing systemically distributed cisplatin. Four cycles of IA infusions are coupled with 2 Gy daily radiation for 5 days per week over 7 weeks for a total of 70 to 74 Gy. Robbins phase 1 dose escalation trial in 1994 established that 150 mg/m^2^ was the maximum tolerated dose of IA cisplatin before significant renal toxicity was observed.^47^ Table 1 shows the history of RADPLAT trials for HNC.

**Table 1:** Summary of Early Single‐Arm RADPLAT Trials 1994–2005

Year	Authors	Study type	Patients (n)	Disease characteristics	Median follow‐ up	Complete initial response (CR)	Partial initial response (PR)	Loco‐regional Control	Survival	Toxicities
1994	Robbins et al.^47^	Phase 1	42	Grade III/IV, 23 untreated, 19 recurrent	15.5 mo	9 (41%) in naïve, 4 (25%) in recurrent	10 (45%) in naïve, 6 (38%) in recurrent			No III/IV toxicities at 150 mg/m^2^
1994	Robbins et al.^49^	NRC	29	HNSCC, IV, all untreated.	22 mo	23 (96%)			Overall: 88% Disease free: 53%	III/IV in 13%
1996	Robbins et al.^50^	NRC	42	Resectable 98% III/IV disease	13 mo	36 (86%)	3 (7%)		Overall: 64%	9% III/IV (n=9)
1997	Robbins et al.^51^	NRC	60	T4 or N2/3	18 mo	52 (91%)	4 (7%)	75%	1‐y 67%	III/IV events 42%
2000	Robbins et al.^52^	Retro‐spective	213	III/IV, 66% resectable disease	30 mo	61%	9%	5‐year, 74.3%	Overall: 53.6% 5‐y: 38.8%	39% experienced a III/IV toxicity
2004	Balm et al.^53^	Retro‐spective	79	Stage IV,	3.1 y	72 (91%)	3 (3.8%)	2‐y 69%	Overall: 43%	57% Grade III GI (tube feeding), 38% III/IV hematologic, 3 pts Grade V tox or treatment related death
2005	Robbins et al.^54^	Phase 2 RCT	67	HNSCCC, Stage IV	3.9 y	80% (49 pts)		1‐y 65.6%, 2‐y 57.2%	Overall 1‐y 72%, 2‐y 63%	Grade III 44%, grade IV 39%, grade V 3%.
2010	Rasch et al.^55^, ^56^, ^57^, ^58^	Phase 3 RCT	239	Stage IV	7.5 y					

Robbins’ early studies showed promising complete response of 86%–96% and 1‐ and 2‐year survival rates of 64%–67%, however the larger 2000 study (n=213) showed CR of only 61%.^51^, ^52^ With a median follow‐up of 30 months, 5‐year projected overall survival was 38.8%. In these early studies, patients received unilateral IA cisplatin infusion, however they noted that in select patients (eg, those in whom the tumor crossed midline) bilateral ECA catheterization and infusion was used. In 2004, Balm et al showed initial response of 91% in patients with stage IV disease and overall 3‐year survival of 43%.^53^ These promising findings prompted the National Cancer Institute to fund a multi‐center phase II trial conducted under the auspices of the Radiation Therapy Oncology Group (RTOG 9615).^54^ Sixty‐seven patients were enrolled at 11 different centers and demonstrated 80% complete response and 2‐year survival of 63.4%.

In 2010, a Dutch group led by Rasch published the only phase 3 randomized trial in 239 patients with stage IV squamous cell carcinoma of the oropharynx, oral cavity, and hypopharynx, and compared RADPLAT with conventional IV chemoradiation.^55^ Notably, only patients with tumors deemed anatomically or functionally unresectable (ie, resection would require glossectomy) were included. At 3 years, there was no significant difference between the IA and IV arms based on locoregional control, disease‐free survival, disease‐specific survival, distant metastasis survival, or overall survival. In the subsequent years, Rasch et al have published multiple follow‐up studies on the Dutch trial focusing on quality of life. Patients treated with IA therapy had significantly less nausea and vomiting in the short term and were less fatigued, but the two arms were otherwise undistinguishable in long term QOL.^56^, ^57^ In 2015, the group published new survival and disease control data based on an extended median follow‐up of 7.5 years. They reported no difference between treatment arms in loco‐regional control or overall survival.^58^

These results diminished excitement around IA chemo for HNSCC, but there are significant limitations of the trial that call into question the negative results.^59^, ^60^ The Dutch trial selected patients with unresectable disease, the majority crossing midline which required bilateral infusions (and therefore potential drug dilution), and 60% with oropharyngeal cancer. A subgroup analysis of 26 patients who received unilateral infusion for large (>30 mL) tumors showed a significantly higher locoregional control rate and disease‐free survival. These parameters are in sharp contrast to prior studies which included patients with resectable disease. Bilateral IA infusion was inferior to unilateral infusion and raises the question of a dilutional effect when the same total dose is given. Additionally, OP lesions are considered more susceptible to chemoradiation and human papilloma virus (HPV) is known to be the primary driver of these lesions. Both of these aspects confer a prognostic advantage,^61^ and as HPV status was not tested in the Dutch trial, this may have masked benefit in less treatable tumors.

In addition to disease control and survival outcomes, there are also important potential benefits for treatment adherence, locoregional control, and treatment‐related toxicity profiles that merit consideration for IA chemotherapy in HNSCC. Receiving treatment for HNC is a challenging experience for patients and treatment protocols are often altered due to patient tolerance. Though patients receiving RADPLAT require several procedures, the potential benefits of reduced systemic toxicities may lead to increased adherence. The studies by Robbins and Balm indicated that >90% of patients received the full course of chemotherapy^51^, ^53^ The larger multi‐institutional trial (RTOG 9615) and Dutch trial both showed ∼75% adherence to the full treatment protocol.^54^ Overall, the data suggest that adherence to RADPLAT is very high and at least as good as adherence to conventional therapy.

Expected toxicities associated with cisplatin, such as renal, hematologic, mucosal, otologic, cardiac, and pulmonary sequelae, are the major dose‐limiting factors. The studies by Robbins et al reported grades III and IV toxicities ranging from 9% to 42%, but there was a virtual absence of grade IV/V nonhematologic toxicities.^49^, ^50^, ^51^, ^52^ This was a major contrast to conventional chemoradiation trials.^51^ A retrospective review of IA chemotherapy by Gemmete et al noted that of the 10.6% rate of grade III/IV chemotoxic events, 71% were mucosal‐related.^62^ However, a small subset of patients experienced access‐site complications and cerebrovascular events at rates comparable to those of other angiographic procedures. It is plausible that the recent broad implementation of radial approach for CNS angiography may help to reduce access‐site complications, though cerebrovascular events will likely remain the major periprocedural complication for these procedures, albeit at a low rate. The Dutch trial showed that the incidence of renal toxicities (> grade 2) was significantly lower in the IA arm compared with the conventional IV arm (1% versus 9%; *P*<0.0001), but this was offset by the fact that neurological toxicity was more prevalent in the IA arm, though no permanent neurological deficits were seen (8 versus 1 patients with TIAs/CVA; *P*=0.005).^55^ There was no significant difference between the two treatment arms for other types of toxicity.

In the setting of IA chemotherapy to improve targeted dosing, concerns for inadequate treatment of disease outside the target vascular territory have been largely unsubstantiated. The studies outlined above report a range of locoregional control from 57% to 74% and were comparable to conventional chemotherapy rates.^52^, ^53^, ^54^ All studies included patients with advanced stage HNC including those with and without nodal involvement. When assessing the role of RADPLAT in locoregional disease control, it is informative to review data for the subgroup of patients with known nodal involvement, which demonstrate only 28.6% residual disease on subsequent neck dissection after chemoradiation and 3‐year 77% locoregional control.^63^, ^64^ At 3 years, locoregional control rate was 67%. These data suggest that despite the targeted mechanism of delivery used in RADPLAT, the effects of the therapy are not strictly limited to the tumor bed.

There is substantial evidence favoring IA RADPLAT as safe and efficacious compared with conventional chemoradiation for advanced HNCs, but the field has yet to demonstrate therapeutic superiority over conventional chemoradiation. Further study is clearly needed, specifically in unilateral disease in HPV‐negative patients. Homma et al are performing a phase III trial in the context of locally advanced maxillary sinus cancer (JCOG 1212),^65^ based on results from NCT00304278 wherein RADPLAT was combined with erlotinib, an EGFR inhibitor, demonstrating feasibility and suggestion of improved outcomes.^66^ Biologics, such as small molecule kinase inhibitors and immunotherapy may also be combined with RADPLAT to provide novel combination therapies. In particular, cetuximab, a monoclonal antibody targeting EGFR‐expressing tumor cells, has been shown to improve survival when added to cisplatin‐fluorouracil in recurrent or metastatic SCCHN.^67^ Cetuximab, when added to chemoradiation, also showed promise in the context of advanced unresectable SCCHN.^68^

**Head and Neck Highlights**

- There is substantial evidence favoring IA chemotherapy as safe and efficacious for advanced HNCs, but therapeutic superiority over conventional chemoradiation has not yet been established. Many studies had limitations such as including large numbers of patients with unresectable midline tumors necessitating bilateral infusion, which may dilute drug delivery, and failing to account for HPV status, which are more sensitive to chemoradiation.
- With emerging therapies and new techniques for intraarterial access and delivery, a renewed focus on IA chemotherapy for head and neck tumors is warranted.

## IA Chemotherapy for Retinoblastoma

Retinoblastoma currently carries the most compelling evidence for neurointerventional IA chemotherapy treatment. Retinoblastoma is the most common primary intraocular malignancy in pediatric patients, with 11% of retinoblastoma cases occurring within the first year of life.^69^, ^70^ Worldwide, approximately 8000 children develop retinoblastoma annually with a prevalence of 11.8 million children diagnosed before the age of 5.^71^, ^72^ Retinoblastoma is a curable cancer if detected early and treated aggressively. In developed countries, survival rates are close to 100%, but this falls to less than 50% in developing countries.^69^, ^73^ However, despite high survival rates, treatment carries significant morbidity, with standard treatments including enucleation and lengthy IV chemotherapy.^74^ In the 1950s, external beam radiation therapy (EBRT) was the first globe‐ and vision‐sparing therapy for advanced retinoblastoma demonstrated to control tumor growth. However, radiation increased the risk of secondary malignancy in children carrying the commonly found RB1 mutation.^75^, ^76^ IV chemotherapy was introduced in 1953 with a triple‐drug therapy of vincristine‐etoposide‐carboplatin performed for 6 cycles with an interval of 28 days.^77^ While initially used as primary treatment, this regimen only reduced tumor size, requiring follow‐up with local surgical treatment.^78^, ^79^, ^80^ This use of chemotherapy is termed chemoreduction and usually requires 2 cycles (12 months) followed by local treatment methods such as cryotherapy, laser transpupillary thermotherapy, and plaque radiotherapy.

Retinoblastoma is classified by size and tumor seeding according to the International Classification of Retinoblastoma: Group A <3 mm in size; Group B >3 mm or macular or juxtapupillary location; Group C‐ subretinal and/or vitreous seeds <3 mm from the retinoblastoma; and Group D‐ subretinal and/or vitreous seeds >3 mm from the retinoblastoma.^81^ Shields et al evaluated the efficacy of chemoreduction followed by local therapy to avoid enucleation and EBRT in a cohort of 249 eyes. They showed 100% resolution in Group A, 93% in Group B, 90% in Group C, and 47% in Group D.^82^ While these were promising results in low grade retinoblastoma, there was poor response in patients with more advanced retinoblastoma. Systemic chemotherapy with focal adjunctive therapy such as transpupillary thermotherapy and cryotherapy has been standard of care for intraocular retinoblastoma for over 40 years.^83^, ^84^, ^85^, ^86^, ^87^, ^88^, ^89^, ^90^

Reese et al first introduced IA chemotherapy for treatment of retinoblastoma in 1958 through the cervical carotid artery with concomitant EBRT. However, this study was halted due to increased systemic toxicity.^75^ In 1984, Kaneko et al studied high dose IA melphalan infused into the ipsilateral ophthalmic artery as the most optimal chemotherapeutic agent to reduce systemic toxicity. In their protocol, a balloon was placed distal to the ophthalmic artery to occlude the internal carotid artery, and melphalan was delivered indirectly into the ophthalmic artery. Subsequently, this technique was refined to place a microcatheter directly into the ophthalmic artery.^83^, ^84^ In 2006, Gobin et al used this modified technique of selective ophthalmic artery catheterization for primary treatment of using IA melphalan with 98.5% technical success and an ocular event‐free survival rate in 81.7% in previously untreated retinoblastomas.^85^ Abramson et al additionally reported efficacious and reproducible results of this novel treatment now termed “super‐selective chemosurgery.”^85^, ^86^ Unlike systemic volume of distribution dosing for IV melphalan, dosing for IA treatment of retinoblastoma is typically based on the size and volume of the eye. In unilateral retinoblastoma, Peterson et al reported that a neoadjuvant IA dose of 7.5 mg melphalan was associated with a lower enucleation rate (0% versus 36%),^87^^,^ 88 and 6 mg resulted in no cases of periorbital edema and neutropenia.^88^ For bilateral retinoblastoma, IA melphalan can be delivered to both eyes, called “tandem therapy.”^86^ Peterson et al calculated a maximum patient dose of 16 mg/m^2^, and for each patient the maximum systemic dose was less than 15 mg total, with minimal adverse effects.^87^ Table 2 shows the history of IA chemotherapy trials for retinoblastoma.

**Table 2:** Relevant Studies on IA Chemotherapy for the Treatment of Retinoblastoma

Year	Author	Study type	Eyes (n)	Disease severity	F/u (month)	Complete initial response	Partial initial response (PR)	Eye salvage (%)	Survival	Toxicity
1958	Reese et al^97^	Prospective	195	Primary IA=18 Salvage IA=13	**3**	24%	12%	60	36%, 12% with limited vision	Not reported
1998–2007	Suzuki et al^103^	Retro‐spective	408	N=143 unilateral, n=209 bilateral Group A=5 Group B=130 Group C=30 Group D=216 Group E=18	74			60	70.0% at 2 ye all eyes, 82% primary IA chemo, 58.4% previously treated	0.5%
2006–2010	Gobin et al^85^	Prospective	95	Primary IA: 43 Salvage IA: 52 Group 1‐3: 8 Group IV: 4 Group Va: 10 Group Vb: 73		91%			76.5%	11.4%
2006–2010	Peterson et al^87^	Prospective	17	Primary IA: 0 Salvage: 17	8.6	53.8%	46.2%			11.5%
2008–2010	Muen et al^93^	Prospective	15	Salvage IA	8.7	80%			59%	47%
2010–2013	Ong et al^107^	Retro‐spective	17	Primary IA n=6, Salvage IA n=11 Group B=3 Group C=1 Group D=1 Group E=12	22	71%	66%			41%
2010–2017	Ammanuel et al^100^	Retro‐spective	43	Primary IA: 0 SalvageIA: 43 Group A: 1 Group B: 10 Group C: 11 Group D: 13 Group E: 8		67.4%	11.6%		96%	1.6%
2009–2017	Rojanaporn et al^90^	Retro‐spective	27	Primary IA: 7 Salvage IA: 20 Group B: 3 Group C: 1 Group D: 12 Group E: 11	32	57%		52	80% after 18 mo, 69% after 30 mo	44%
2012–2017	Lumbroso et al^106^	Prospective	39 patients, 39 eyes	Primary IA: 39 Salvage IA: 0 Group B: 5 Group C: 13 Group D: 21	63	75%	25%	74.4	100%	21%

IA, intraarterial.

IA delivery of chemotherapy for retinoblastoma is typically an outpatient procedure under general anesthesia due to the young age of this patient population. The ipsilateral femoral artery can be accessed using a 4‐French pediatric arterial sheath with selective catheterization of the ophthalmic artery with a 0.008–0.010‐inch microcatheter and microwire. The microcatheter can usually be wedged in the origin of the ophthalmic artery. Microangiography should be performed to ensure that contrast flows solely into the ophthalmic artery and not distally into the ICA to prevent drug dilution or neurotoxicity. Stenzel et al, reported technical challenges including difficulty cannulating the ophthalmic artery in 8.2% of cases and anatomical variants with meningeal collaterals in 12.2% of patients with an alternative blood supply of the retina in 9.2% of patients.^89^ If ophthalmic artery catheterization is technically successful, the patient is heparinized with 70 to 80 units/kg IV to prevent thromboembolic complications. The calculated dose of melphalan is diluted in 30 mL of normal saline and slowly infused over 20 to 30 minutes in a pulsatile fashion. In bilateral retinoblastoma, this is repeated in the contralateral ophthalmic artery and the total dose is adjusted between the two sides to avoid cumulative toxicity. Patients are admitted for observation for 4–6 hours and are discharged the same day with topical steroid eye drops and short acting mydriatics. Oral aspirin (1–2 mg/kg body weight) may be used for 2 weeks postoperatively as an option, though not universally used. Patients are advised to return in 7–10 days for a complete blood count.^90^ Transpupillary thermotherapy may be planned in combination with IA chemotherapy, which can be performed through indirect ophthalmoscopy through a dilated pupil with a 1.2 mm spot size iris diode laser beam (810 nm) set at approximately 350 mW. Laser is applied continuously until clear whitening occurs in the tumor and/or retina for synergy of the heat energy and chemotherapeutic to improve efficacy.^91^

When compared with IV chemotherapy, IA chemotherapy showed significantly higher globe salvage in patients with advanced retinoblastoma (Group D), but showed no statistically significant difference in treatment outcomes for Group A‐C eyes or metastasis rates among both cohorts.^92^ IA chemotherapy demonstrated a risk of local complications due to the high local concentration of drug, though usually mild and self‐limited such as blepharoptosis, forehead hyperemia, and eyelid edema.^93^ IA chemotherapy demonstrated a 95% and 100% tumor regression rate for small and medium sized tumors, respectively, which is comparable to IV chemotherapy.^94^ In advanced intraocular retinoblastoma that would have otherwise required EBRT or enucleation, IA chemotherapy offers a 75% eye salvage rate.^94^ In a systematic review, Berry, et al. reported a 0%–23% tumor recurrence after IA chemotherapy, compared with 35%–45% after isolated IV chemotherapy and 17%–18% after IV chemotherapy combined with focal treatment methods.^95^ IA chemotherapy was proposed to help mitigate systemic toxicity with the combined use of focal therapy, particularly laser transpupillary thermotherapy, to boost outcomes.^96^ Francis et al reviewed 452 eyes with retinoblastoma treated with IA chemotherapy and found a one‐year recurrence rate of 25%, with half the recurrences necessitating only focal methods (laser, cryotherapy, brachytherapy, intravitreous chemotherapy) for retreatment.^97^ Chen et al also reported the successful elimination of recurrent tumors in 8 of 29 eyes treated with salvage IA chemotherapy.^98^ There has been a resultant paradigm shift that IA chemotherapy for retinoblastoma should now be considered a first‐line treatment of unilateral and bilateral retinoblastoma over EBRT, enucleation, or IV chemotherapy.^94^

The major advantage of IA chemotherapy is reduction of systemic toxicity of chemotherapeutic agents. IV chemotherapy causes myelosuppression, cytopenia, or neutropenia in as many as 100% of patients, the need for red blood cell transfusions in 60% of patients, platelet transfusions in 48%, and febrile neutropenia in 50% of patients.^99^ With IA therapy, neutropenia was reported with IAC in 5.9% with self‐resolving complications such as periocular edema and hyperemia (34%), ptosis (13.5%), focal madarosis (10.5%), and forehead erythema (3%).^94^ IA treatment is associated with self‐resolving orbital cranial nerve palsy, reported in up to 35%,^93^ however only 3 cases of stroke following IAC have been reported.^100^, ^101^ Vision‐threatening complications associated with conditions such as central retinal artery occlusion, vitreous hemorrhage and retinal detachment are rare theoretical risks;^94^ however, comparing complications from the 2009 to 2011 time period to 2012 to 2017, Dalvin et al, reported a reduction of vascular complications from 59% to 9% and choroidal ischemia from 14% to 4%.^102^ Again, the local nature of IA chemotherapy raises concern for lack of treatment for undetected systemic metastasis,^103^ however, a multi‐institutional survey of prominent IA chemotherapy centers treating retinoblastoma analyzed metastatic deaths over 10 years. Of 1139 patients treated with IA chemotherapy over this span, only 3 patients were reported to have metastasis.^104^, ^105^ In the United States, Gobin et al, reported 2 of 78 patients who developed metastasis at 13 months.^85^

In summary, retinoblastoma is a highly curable intraocular malignancy in pediatric patients. IA chemotherapy as first or second line therapy for patients with unilateral or bilateral retinoblastoma, especially in higher grade disease, is an efficacious and promising treatment regimen, with 74% ocular preservation at 18 months and 69.3% ocular preservation at 36 months, and minimal adverse events.^106^ Due to the lack of randomized controlled trials in this area, clinical trials are warranted to assess the long‐term impact on patient quality of life and whether IA therapy should become primary standard of care for treatment of retinoblastoma.

**Retinoblastoma Highlights**

- Retinoblastoma is one of the most common curable primary intraocular malignancies in pediatric patients, but requires IV triple‐drug regimen of vincristine‐etoposide‐carboplatin in addition to enucleation for advanced disease.
- Selective IA Melphalan for treatment of high‐grade retinoblastoma results in 95%–100% tumor regression rate, limited recurrence or metastatic disease, and is associated with a significantly *lower* enucleation rate and systemic side effects.
- Selective IA Melphalan should be considered for high‐grade retinoblastoma.

## IA Chemotherapy for Glioblastoma and Other CNS Tumors

Glioblastoma multiforme (WHO grade IV astrocytoma, GBM), is the most common malignant brain tumor, accounting for nearly 50% of malignant brain tumors. It has an incidence of 3.21 per 100 000, with a 1‐year survival of 40%, and a 2‐year survival of 17%.^108^ The Stupp protocol is the current standard of care treatment of GBM,^109^ which includes 60 Gy of radiation given in 2 Gy fractions daily, 5 days per week for 6 weeks, and temozolomide given at a dose of 75 mg/m^2^ of body‐surface area per day, 7 days a week during radiotherapy, followed by 6 adjuvant cycles consisting of 150–200 mg/m^2^ for 5 days during a 28 day cycle.^109^ Chemotherapy for intracerebral tumors is limited by the BBB. Most chemotherapeutic agents cannot reach sufficient levels of penetration in CNS tissues unless they can permeate the BBB, or unless the BBB can be temporarily disrupted to allow sufficient passage of these agents into the CNS tissues to achieve efficacy. This effect contributes to poor survival rates of high grade astrocytomas and glioblastoma multiforme despite adjunctive therapies.

Initial pilot studies of rescue IA cisplatin for malignant glioma were unsuccessful.^110^ Direct delivery of IA cisplatin and etoposide into the tumor bed prior to radiotherapy, however, has shown significant treatment advantage when compared with different chemoradiation treatment modalities, with a posited mechanism of etoposide‐mediated BBB disruption.^111^ Additionally, the patients in this trial were treatment naïve to chemotherapy or radiation therapy and differing pharmacokinetics in these patients may also have played a role in improved outcomes. Patients with high grade astrocytoma receiving IA chemotherapy prior to whole brain radiation therapy (WBRT) were compared to concomitant IA chemotherapy with WBRT.^111^ The first group received IA infusion of cisplatin with etoposide every 3 weeks followed by WBRT, and the second group received IA infusion of cisplatin with etoposide every 3 weeks while undergoing concomitant WBRT. Dexamethasone for cerebral edema, initiated 2 weeks prior to treatment, was used in both groups. Interestingly, the patients receiving WBRT after IA chemotherapy had a survival advantage compared with those receiving concomitant IA chemotherapy + WBRT (median survival of 20‐months versus 7‐months for GBM and 45 months versus 12 months for anaplastic astrocytoma, respectively). Minimal side effects were observed, with no fatalities or cognitive toxicities. It is postulated that WBRT prior to or in combination with IA chemotherapy may negate the positive effects of IA chemotherapy by jeopardizing the tumor vasculature, leading to decreased chemotherapeutic concentrations within tumor tissue.^112^ Multiple additional studies including studies by Imbesi et al and Kochi et al have compared the effectiveness of IA versus IV nimustine (ACNU) for the treatment of newly diagnosed GBM. Both studies found no advantage in progression‐free survival or overall survival when comparing to patients treated with IV ACNU.^113^, ^114^, ^115^

More recently Patel et al, have evaluated the efficacy of IA bevacizumab after disruption of the BBB for treatment of newly diagnosed glioblastoma.^116^ Twenty‐three patients underwent surgical resection or biopsy followed by sequential IA administration of a standard dose of 20% mannitol (12.5 mL delivered over 120 seconds) for disruption of the BBB and then 15mg/kg IA bevacizumab after 30, 120, and 210 days delivered directly to blood vessels supplying the tumor. Clinical follow‐up was obtained at 1 month with MRI performed every 2 months. Patients underwent concurrent temozolomide treatment per the Stupp protocol.^109^ Median progression‐free survival was found to be 11.5 months, defined as no increasing enhancement on T1 post‐contrast imaging or increasing mass like hyperintensity on T2 FLAIR imaging, and no development of new lesions. Progression‐free survival was 91.3% at 6 months, 47.4% at 12 months, 32.5% at 24 months, and 5.4% at 5 years. Median overall survival was 23.1 months, with a 5‐year survival rate of 16.1%. Additionally, no substantial complications were seen. This study is confounded by the lack of control group, but their treatment outcomes are encouraging when compared to prior GBM studies. For comparison, the current standard of care, the Stupp protocol, carries a 26.5% overall survival at 24 months.^109^

IA chemotherapy has also been investigated for recurrent GBM.^117^, ^118^ Small studies of IA infusion of bevacizumab after BBB disruption by IA mannitol infusion showed progression‐free survival of 10 months, and overall survival 8.8 months, though 4 patients in the cohort died before progression was seen. Patients were found to have few complications. IA bevacizumab had statistically significant decrease in tumoral enhancement after 1 month following treatment, with progression‐free survival of 10 months, when compared with prior studies for patients treated with IV bevacizumab alone which showed progression‐free survival rates of 3.7^119^ and 4.2^120^ months, and overall survival similar to prior studies which were 7.2^119^ and 9.2^120^ months, as compared with this study's overall survival of 8.8 months. Table 3 shows the history of IA chemotherapy trials for GBM.

**Table 3:** Key Studies of IA Chemotherapy for the Treatment of CNS Tumors

**Year**	**Author**	**Study type**	**Disease type**	**Type of therapy**	**Pts (n)**	**CR %**	**PR %**	**Progression‐free survival**	**Overall survival**	**Toxicity**
2000	Madajewicz et al.^111^	Single center, Pro‐spective, Phase II	High grade Astro‐cytoma	IA cisplatin + etoposide before WBRT vs IA cisplatin + etoposide during WBRT	71	5.6 vs 0%	28.2% vs 14.1%	NR	20 mo GBM, 45 mo anaplastic vs 7 mo GBM, 10 mo anaplastic	N/V 14%, transient confusion 1.8%, seizure 1.4%
2021	Patel et al.^116^	Single center, Prospective, Phase I/II	Newly diagnosed GBM	IA bevacizumab + concurrent Temozolomide	23	NR	Not reported (NR)	11.5 mo	23.1 mo	No grade III/IV toxicities
1989	Newton et al.^110^	Single center, Pro‐spective, Phase I/II trial	Recurrent Malignant Glioma	IA cisplatin	12	0%	8.30%	NR	NR	3 Myelo‐suppressio, 2 Retinal toxicity, Sz 2, Death 1, TIA 4
2006	Imbesi et al.^113^	Single center, Pro‐spective RCT Phase III	Newly diagnosed GBM	IA Nimustine vs IV Nimustine	43	0% vs 0%	6.9% vs 0%	6 mo vs 4 mo	17 mo vs 20 mo	3 hematologic, 1 CVA versus 8 hematologic
2000	Kochii et al.^114^	Multi‐center, Prospective RCT Phase III	Newly diagnosed GBM	IA Nimustine vs IV Nimustine + Rtx	82	NR	NR	24 wks vs 45 wks	59 wks vs 56 wks	7 grade III/IV versus 16 Grade III/IV
2011	Burkhardt et al.^118^	Single center, Pro‐spective, Phase II	Recurrent GBM	IA bevacizumab after BBB disruption with Mannitol	14	0%	57.10%	10 mo	8.8 mo	No grade III/IV toxicities
2007	Sonoda et al.^123^	Retro‐spective	PCNSL	IA Nimustine with WBRT	62	75%	25%	26 mo	39 mo	1 grade V toxicity (interstitial pneumonia leading to death), 14 late neurotoxicity
2009	Angelov et al.^124^	Multi‐center, Prospective, Phase II	PCNSL	IA Methotrexate + BBB disruption with Mannitol	149	57.70%	24.20%	1.8 y	3.1 y	Sz 33.6%, CVA 7.4%, Fever 2.8%, DVT/PE 2.6%
2021	Iorio‐Morin et al.^125^	Retro‐spective	PCNSL	IA methotrexate + BBB disruption with Mannitol	44	79.50%	NR	24 mo	45 mo	25 grade III, 8 grade IV

BBB, blood–brain barrier; CNS, central nervous system; GBM; glioblastoma multiforme; IA, intraarterial; PCNSL, primary central nervous system lymphoma; WBRT, whole brain radiation therapy.

### CNS Lymphoma

Primary central nervous system lymphoma (PCNSL) is another CNS tumor for which IA chemotherapy has been investigated. PCSNL accounts for approximately 2% of all central nervous system malignancies. With treatment, 5‐year survival is approximately 30%.^121^ PCSNL has historically been treated with radiation therapy in conjunction with IV methotrexate, resulting in improved survival outcomes, though with a high rate of clinical neurotoxicity, particularly in older individuals.^122^ As with most chemotherapeutic agents, CNS penetration by methotrexate is limited by the BBB. As such, high doses of methotrexate are required to achieve treatment response, resulting in increased risk of renal and neurotoxicity. IA chemotherapy has been investigated as an alternative treatment strategy with or without disruption of the BBB. Sonoda, et al. studied IA ACNU in conjunction with WBRT.^123^ They followed 63 patients with newly diagnosed PCSNL who underwent 36–50 Gy WBRT in 18–25 fractions over 4–5 weeks with a boost to the lesion site or 30 Gy WBRT in 15 fractions over 3 weeks with a boost to the lesion site, in conjunction with IA ACNU. Of 62 patients who completed the protocol, 75% achieved complete response and 25% achieved partial response. Median disease progression‐free survival was 26 months, while overall survival was 39 months. Overall, 3‐ and 5‐year survival following treatment were estimated to be 51% and 32%, respectively, and overall treatment response was 100%, which is comparable to published literature for treatment with methotrexate ± WBRT. A significant complication within this study group was the development of late neurotoxicity, which was found to be more common in patients older than 60 years, and at similar rates as patients undergoing a high‐dose methotrexate regimen, though the authors do note that a component of this effect could be secondary to radiation therapy. The authors conclude that IA ACNU administration combined with WBRT provided good treatment outcomes at acceptable toxicity levels for patients younger than 60 years and suggest an alternative regimen for those older than 60, which includes IA ACNU administration, with a decreased or delayed dose of radiation therapy. It is possible that, similar to the GBM study by Madjewicz et al, IA chemotherapy prior to WBRT may yield superior results due to intact tumor vasculature, allowing the chemotherapy to access the tumor more effectively.^111^

Subsequent studies have explored the use of IA methotrexate administration with disruption of the BBB to improve the distribution of methotrexate within the central nervous system. One hundred forty‐nine patients with newly diagnosed PCNSL were treated at 4 institutions with disruption of the BBB with 25% IA mannitol (6–12 mL/sec over 30 seconds), 5 minutes after which a 15‐minute IA infusion of methotrexate (2.5 g) was performed. Methotrexate was given for 2 consecutive days every 4 weeks for up to 12 months.^124^ Etoposide (150 mg/m^2^ IV days 1 and 2) and cyclophosphamide (500 mg/m^2^ IV days 1 and 2) were also used in combination with methotrexate. As discussed, etoposide may have a natural ability to disrupt the BBB. Eighty‐six patients (58%) were found to have complete response with no evidence of leptomeningeal disease, ocular lymphoma, or abnormalities of CSF cytology in the absence of corticosteroid use. Thirty‐six (24%) were found to have partial response, for an overall response rate of 81.9%. Median progression‐free survival was 1.8 years with median overall survival of 3.1 years. Survival at 3 and 5 years were 41% and 25% respectively. Of 96 patients who died during follow‐up, 46 succumbed to CNS lymphoma progression, while 9 succumbed to systemic recurrence. Patients >60 years old and those with Karnofsky Performance Scale <70 had lower mean overall survival as well as lower progression‐free survival. Complications from BBB disruption and IA injection itself include seizure and stroke, with a risk of permanent neurological deficit found to be 0.2% per treatment. These results are comparable or even superior to the use of high dose of IV methotrexate alone, and comparable to results of high dose methotrexate combined with radiation therapy, without the sequelae of significant neuro‐cognitive decline.

These findings were corroborated in a smaller study (n=44) using IA 25% mannitol infusion at a dose of 3 to 6 mL over 3 seconds for disruption of the BBB followed by administration of 5g IA methotrexate.^125^ Earlier studies included in the treatment regimen IV etoposide phosphate (400 mg/m^2^) and IV cyclophosphamide (660 mg/m^2^), while more recent studies added IA carboplatin (400 mg/m^2^). Median progression‐free survival was found to be 24 months, and 3‐ and 5‐year survival rates were found to be 57% and 39% respectively. Unlike the findings of Angelov et al, this study cohort did not find that age impacted overall survival or progression‐free survival. One patient developed a permanent deficit from stroke, and one required ICU care secondary to status epilepticus. Currently, the results of trials for treatment of central nervous system malignancies appear to be encouraging, particularly for treatment of newly diagnosed GBM naïve to prior treatment, and primary central nervous system lymphoma.

**GBM and Other CNS Tumor Highlights**

- The BBB inhibits many chemotherapeutic agents from reaching sufficient levels in intracerebral tumors.
- Studies involving disruption of the BBB with IA mannitol, followed by IA chemotherapeutic agents such as bevacizumab or cisplatin+etoposide, showed treatment advantage (prolonged overall survival, prolonged progression‐free survival) when compared with standard of care treatment for glioblastoma multiforme.
- WBRT performed following IA chemotherapy showed treatment advantage when compared with WBRT performed concomitantly with IA chemotherapy, likely due to to WBRT decreasing tumor vasculature thereby limiting chemotherapeutic penetration.
- PCNSL accounts for 2% of CNS malignancies and is historically treated with radiation and IV methotrexate, which often results in toxicity at doses required to penetrate the BBB. IA methotrexate after disruption of the BBB with IA mannitol have shown similar or superior treatment outcomes to standard of care, with decreased rates of neuro‐toxicities.

## Procedural Considerations

IA chemotherapy infusion uses standard angiographic set‐up and procedure, with guide catheter size dependent on patient age and anatomy. Often, 5F is sufficient, using either a standard diagnostic catheter or a large bore 5F, such as an Envoy, in order to perform follow‐up angiography during the infusion. We have adopted a radial‐first approach, to decrease periprocedural risk. The procedure for infusion is well‐known and often utilized in the neurointerventional realm for treatment of vasospasm, thrombolytics in stroke, etc. An 010″ to 021″ microcatheter may be used for infusion; we do not find added benefit from microcatheters larger than this which create increased dead space. There is no evidence yet to support injector infusion over hand infusion, but we prefer pulsatile hand infusion.

There are some key lessons learned in studying past trials of IA chemo. *Selective* catheterization for directed infusion as close to the tumor bed as possible is critical to prevent toxicity to other tissues, such as in selective ophthalmic artery catheterization. A microangiographic run through the microcatheter should be performed to confirm the flow pattern. Selective infusion also prevents drug dilution into the systemic space rather than the tumor bed. From the RADPLAT studies for head and neck cancers, there did seem to be a lesser effect when the dose was split and infused bilaterally. Whenever possible, it is best not to dilute the drug effect. After full infusion of the drug over 20 to 30 minutes, the catheter should be fully flushed, removed, and disposed of in special chemotherapy disposal containers. All infusions should be performed in collaboration with the oncology department to ensure best multi‐disciplinary care and practices and in accordance with hospital chemotherapy protocols, which may differ from typical neurointerventional protocols.

Another important finding from past studies is complication avoidance. The most severe complication of IA chemo is stroke, and full heparinization is highly recommended to precent thromboembolic complications, as the catheter will be in place for about 20 to 30 minutes during the infusion. With the advent of transradial interventions, the risk of groin‐site hematomas/pseudoaneurysms is lessened and allows for adequate anticoagulation.

IA chemotherapy prior to WBRT may yield superior results due to intact tumor vasculature, allowing the chemotherapy to access the tumor more effectively, as seen in GBM trials.^111^ This benefit may be lost after tumors have undergone radiation, which may impact efficacy, thus IA chemo infusions should be done prior to radiation.^111^

Finally, CNS tumors likely require some form of BBB breakdown, with either pre‐treatment with IA mannitol (25% IA mannitol, 3–10 mL/sec over 30 seconds, or 20% IA mannitol, 12.5 mL over 120 seconds), or the use of etopside as a chemotherapeutic which naturally breaks down the BBB. These are examples of several lessons learned, but as previously stated, robust investigation into the IA pharmacokinetics will be imperative as we develop this new treatment modality.

## Future Directions

Though IA chemotherapy has been successfully adopted for some tumors (eg, hepatic carcinomas)^17^ and has demonstrated feasibility with signals for superiority in others (eg, retinoblastoma, glioblastoma, lymphoma), there are still numerous areas for improvement and novel areas which may benefit from this approach. For example, IA drug administration may be ideal for the delivery of novel or emerging therapeutics such as immunotherapy, which is quickly becoming a first‐line therapy for numerous types of cancer. Melanoma, lung cancer, and head and neck cancers (HNC) are examples in which immunotherapy has clinically proven efficacy.^126^, ^127^ As an example, pembrolizumab is a PD‐1 antibody that disrupts the PD‐1/PDL‐1 interaction between cancer cells and immune cells and thereby prevents transmission of an immune‐inactivating signal. It is traditionally given by IV infusion. The drug's use in a clinical setting is well documented and has led to favorable outcomes in a significant number of patients.^128^ Favorable preclinical results for immunotherapy as a treatment for glioblastoma have been published, but clinical studies have thus far been disappointing.^129^

Given the mechanism of action, it is reasonable to anticipate that local delivery of immunotherapy would stimulate a local immune response via draining lymph nodes and may allow more direct control of the local drug concentration. IA delivery of various types of immunotherapies has recently been evaluated in a pre‐clinical setting, and there is at least one clinical trial evaluating IA pembrolizumab.^54^, ^130^, ^131^ These studies indicate that similar or greater efficacy of the drug is achievable with local delivery. There are currently no documented studies of IA immunotherapy for the treatment of brain tumors. The theoretically reduced systemic exposure could translate to reduced number of cycles and therefore side effects, while improving local response. The scientific rationale for directly stimulating a local immune response compliments the promise of reduced systemic exposure and reduced adverse effects. Collectively, IA delivery of immunotherapy warrants further clinical investigation against all types of cancer, particularly HNCs and glioblastoma.

One purported drawback of IA administration is the ability to control metastatic disease that may be outside of the primary tumor location. Studies regarding RADPLAT have demonstrated good locoregional control, as discussed above. It may be that once the primary tumor is controlled with IA chemotherapy, the immune system could be primed to target residual disease. Supplemental administration of shorter systemic regimens could be given to target microscopic residual disease. Targeting metastatic disease is less essential for primary tumors in the CNS as these do not migrate systemically, though targeting spread within the CNS is important. Nonetheless, this will be another important parameter to address for IA chemotherapy. Finally, it is essential to note that IA drug delivery must ultimately show some proven clinical benefit, whether it be related to treatment efficacy, side effect profile, or some combination thereof to outweigh the fiscal and treatment‐related potential risk profile. As discussed above, there are inherent risks to IA delivery that do not exist with IV delivery, not least of which is the potential for a cerebrovascular accident. Additionally, IA therapy is relatively more invasive than its IV counterpart and each procedure requires time and resources. However, performing the procedure using trans‐radial access may further decrease the procedural risk. For these reasons, a clear clinical benefit, one that outweighs these risks, must be demonstrated to solidify IA chemotherapy as a standard first line or salvage treatment option.

## Considerations for Future Clinical Trials

When planning clinical trials, it is imperative to avoid pitfalls that may obscure the benefit of IA drug delivery. As with all clinical trials, powered studies in which the patients are appropriately randomized is an important first step. Beyond this, having matched patients with similar disease burden and tumors that have similar molecular characteristics are very important to make direct comparisons between IA and standard delivery routes. One such example is HPV as the genetic driver of most oropharyngeal cancers. In the Dutch clinical trial targeting head and neck cancers described above, HPV status was unknown. Therefore, any discrepancies between treatment groups were also unknown, and this calls into question the results of the trial. This will likely come into play for GBM and possibly PCNSL. For trials targeting CNS tumors, utilizing the strategies outlined above to maximize BBB penetration will also be important and must be considered when designing trials, and close attention should be paid to the order of radiation and IA infusion, as prior radiation may diminish the tumor vasculature and render IA chemotherapy less successful.

Studying focused and similar patient populations with highest chance of success based on previous trials is important as we begin to test for superiority over conventional IV therapies. In addition to identifying ideal IA chemotherapeutic drug choices, we will need to establish clear pharmacokinetics, local tumor and systemic tissue effects, and quality of life measurements. Much can be learned from the randomized RADPLAT trials to help design future Phase III trials for retinoblastoma, GBM, and PCNSL.

## Conclusion

With a preponderance of feasibility data, and signals of efficacy for treatment of CNS neoplastic disease, head and neck tumors, and retinoblastoma, as well as novel therapeutics, IA chemotherapy is a robust and exciting area of research and prospective clinical trials involving the neurointerventional community are warranted. Trials from head and neck cancers show biologic plausibility for IA chemotherapy with signals for clinical benefit in properly selected populations. Retinoblastoma currently carries the most compelling evidence for neurointerventional IA chemotherapy treatment, especially in more advanced disease that would otherwise require EBRT or enucleation, with a 75% eye salvage rate. IA bevacizumab for newly diagnosed GBM shows promise, with progression‐free survival 47.4% at 12 months, and 32.5% at 24 months, and IA regimens for PCNSL are comparable or even superior to the use of high dose of IV methotrexate alone. We look forward to well‐designed clinical trials of IA chemotherapies in the neurointerventional space.

## Sources of Funding

None.

## Disclosures

None.

## Supporting information

Supporting Information
