# Supplementary material for: Current Indications for Intraarterial Chemotherapy in Neurointerventional Surgery
Source: Stroke Vasc Interv Neurol. 2022 Nov 7;3(2):e000425. doi: 10.1161/SVIN.122.000425 (PMC12778610; doi:10.1161/SVIN.122.000425)
Supplement: Supplementary file 1 — Supporting Information [file SVI2-3-e000425-s001.docx]

Supplemental Material- Table 1

**Table 1:** Current Clinical Trials using IA Interventions for Treatment of Solid Tumors

| Title | Conditions | Trial ID |
| --- | --- | --- |
| Adjuvant Intraarterial Chemotherapy Following Surgery to Treat Locally Advanced Bladder Cancer | Bladder Cancer | [NCT01627197](https://clinicaltrials.gov/show/NCT01627197) |
| Hepatic Arterial Infusion of Gemcitabine-oxaliplatin for Second-line Therapy in Non-metastatic Unresectable Intra-hepatic Cholangiocarcinoma | Cholangiocarcinoma | [NCT03364530](https://clinicaltrials.gov/show/NCT03364530) |
| Downsizing of Unresectable Cholangiocarcinoma by Combined Intravenous and Intra-arterial Chemotherapy | Cholangiocarcinoma | [NCT01692704](https://clinicaltrials.gov/show/NCT01692704) |
| Biliary Drainage Plus HAIC in Locally Advanced pCCA | Cholangiocarcinoma | [NCT05024513](https://clinicaltrials.gov/show/NCT05024513) |
| Hepatic Arterial Infusion Plus Chemotherapy in Treating Patients With Colorectal Cancer Metastatic to the Liver | Metastatic CRC | [NCT00026234](https://clinicaltrials.gov/show/NCT00026234) |
| Codman Catheter/Synchromed Pump Hepatic Artery Chemotherapy for Unresectable Colorectal Metastases/Intrahepatic Cholangiocarcinoma | Metastatic CRC | [NCT04276090](https://clinicaltrials.gov/show/NCT04276090) |
| Observational Study on Second Line Treatment of Liver Metastases With DEBIRI and Cetuximab | Metastatic CRC | [NCT01891552](https://clinicaltrials.gov/show/NCT01891552) |
| Selective Internal Radiation Therapy (SIRT) in Patients With Unresectable Colorectal Cancer Liver Metastases Who Failed Prior Intraarterial Pump Chemotherapy | Metastatic CRC | [NCT00972036](https://clinicaltrials.gov/show/NCT00972036) |
| Chemoembolization (Lifepearls-Irinotecan) in Patients With Colorectal Cancer and Metastatic Disease | Metastatic CRC | [NCT04595266](https://clinicaltrials.gov/show/NCT04595266) |
| TKM 080301 for Primary or Secondary Liver Cancer | CRC | [NCT01437007](https://clinicaltrials.gov/show/NCT01437007) |
| Intra-arterial Hepatic Beads Loaded With Irinotecan With Concomitant Chemotherapy With FOLFOX in Patients With Colorectal Cancer With Unresectable Liver Metastases: a Phase II Multicenter Study | Metastatic CRC | [NCT01839877](https://clinicaltrials.gov/show/NCT01839877) |
| Hepatic Arterial Chemotherapy With Raltitrexed and Oxaliplatin Versus Standard Chemotherapy in Unresectable Liver Metastases From Colorectal Cancer After Conventional Chemotherapy Failure | Metastatic CRC | [NCT01348412](https://clinicaltrials.gov/show/NCT01348412) |
| HAI-Floxuridine, or Liver-Tx, Combined With 2nd Line Chemotherapy Versus 2nd Line Chemotherapy Alone for Patients With Colorectal Liver metastases and Heavy Tumour Burden. | Metastatic CRC | [NCT04898504](https://clinicaltrials.gov/show/NCT04898504) |
| Combination Chemotherapy in Treating Patients With Colorectal Cancer That Has Spread to the Liver | Metastatic CRC | [NCT00006050](https://clinicaltrials.gov/show/NCT00006050) |
| Leucovorin, Fluorouracil, Cetuximab, and Oxaliplatin in Treating Patients With Stage IV Colorectal Cancer and Liver Metastases That Cannot Be Removed by Surgery | Metastatic CRC | [NCT00544349](https://clinicaltrials.gov/show/NCT00544349) |
| Comparing Hepatic Intra-arterial Injection of Yttrium-90 Microspheres Versus Fluorouracil (5FU) in Colorectal Cancer Metastatic to the Liver Only | Metastatic CRC | [NCT00199173](https://clinicaltrials.gov/show/NCT00199173) |
| Systemic Oxaliplatin or Intra-arterial Chemotherapy Combined With LV5FU2 and an Target Therapy in First Line Treatment of Metastatic Colorectal Cancer Restricted to the Liver | Metastatic CRC | [NCT02885753](https://clinicaltrials.gov/show/NCT02885753) |
| Intra-arterial Hepatic Bevacizumab and Systemic Chemotherapy | Metastatic CRC | [NCT01677884](https://clinicaltrials.gov/show/NCT01677884) |
| Hepatic Arterial Infusion Pump Chemotherapy Combined With Systemic Chemotherapy (PUMP-IT) | Metastatic CRC | [NCT04552093](https://clinicaltrials.gov/show/NCT04552093) |
| Phase II Study Comparing Conversion Rate to Surgery with Hepatic Arterial Infusion Chemotherapy to Systemic Chemotherapy in Patients With Non Resectable Liver-only Colorectal Metastases | Metastatic CRC | [NCT05103020](https://clinicaltrials.gov/show/NCT05103020) |
| Chemoembolization, Irinotecan Bead, Second Line Chemotherapy Treatment of Unresectable Metastatic Colorectal Cancer | Metastatic CRC | [NCT00816777](https://clinicaltrials.gov/show/NCT00816777) |
| Preoperative Intraarterial Chemoembolization Combined with Radiotherapy in Locally Advanced Rectal Cancer | CRC | [NCT03601156](https://clinicaltrials.gov/show/NCT03601156) |
| Comparison of SEEOX and SOX Regimens in Stage IIIB/IIIC Gastric Cancer Patients | Gastric | [NCT02338518](https://clinicaltrials.gov/show/NCT02338518) |
| Intra-arterial Infusion Chemotherapy Combined with Sodium Bicarbonate for Unresectable Gastric Cancer | Gastric | [NCT03822130](https://clinicaltrials.gov/show/NCT03822130) |
| Allogenic Immunotherapy Based on Natural Killer (NK) Cell Adoptive Transfer in Metastatic Gastrointestinal Carcinoma Treated with Cetuximab | Gastrointestinal | [NCT02845999](https://clinicaltrials.gov/show/NCT02845999) |
| Neoadjuvant Chemotherapy with SEEOX Regimen for Borrmann Type 4 Gastric Cancer | Gastrointestinal | [NCT02949258](https://clinicaltrials.gov/show/NCT02949258) |
| Hepatocellular Carcinoma on Cirrhosis with Child A/B7 and Hepatic Intra Arterial Injection of Idarubicin/Lipiodol Emulsion | HCC | [NCT03727633](https://clinicaltrials.gov/show/NCT03727633) |
| Prevention and Treatment of Complications of Endovascular Methods in Patients with Malignant Liver Tumors | HCC | [NCT04764409](https://clinicaltrials.gov/show/NCT04764409) |
| The Impact on Hepatic Recurrence After Adjuvant Chemotherapy with Intraarterial Infusion of Idarubicin-Lipiodol | HCC | [NCT04178642](https://clinicaltrials.gov/show/NCT04178642) |
| Study of Sorafenib in Combination with Low-dose 5-fluorouracil/Cisplatin (FP) Intraarterial Infusion Chemotherapy | HCC | [NCT00933816](https://clinicaltrials.gov/show/NCT00933816) |
| HAIC Versus TACE for Large Hepatocellular Carcinoma Staged BCLC A/B. | HCC | [NCT02973685](https://clinicaltrials.gov/show/NCT02973685) |
| HAIC Versus TACE for Large and Unresectable Hepatocellular Carcinoma Staged BCLC A/B | HCC | [NCT03048123](https://clinicaltrials.gov/show/NCT03048123) |
| Neoadjuvant HAIC for Resectable Hepatocellular Carcinoma Beyond Milan Criteria | HCC | [NCT03469479](https://clinicaltrials.gov/show/NCT03469479) |
| HAIC Plus Sorafenib Versus TACE Plus Sorafenib for Advanced HCC | HCC | [NCT02856126](https://clinicaltrials.gov/show/NCT02856126) |
| The Study of The Treatment of Postoperative Adjuvant Apatinib vs. TACE in Hepatocellular Carcinoma Patients | HCC | [NCT03511703](https://clinicaltrials.gov/show/NCT03511703) |
| TACE Plus PD-1 Antibody vs TACE Alone for Unresectable HCC | HCC | [NCT03782831](https://clinicaltrials.gov/show/NCT03782831) |
| Pharmacological Manipulation of Intrahepatic Arterial Blood Flow in HCC | HCC | [NCT02472249](https://clinicaltrials.gov/show/NCT02472249) |
| Lenvatinib Plus PD-1 Antibody vs TACE for Intermediate-stage HCC Beyond Up-to-seven Criteria | HCC | [NCT03791918](https://clinicaltrials.gov/show/NCT03791918) |
| Outcome of Transarterial Chemo-embolization (TACE) in Hepatocellular Carcinoma Patients With Partial Portal Vein Thrombosis | HCC | [NCT03007212](https://clinicaltrials.gov/show/NCT03007212) |
| HAIC Using Oxaliplatin Plus Fluorouracil/Leucovorin for Patients with Locally Advanced HCC | HCC | [NCT02436044](https://clinicaltrials.gov/show/NCT02436044) |
| A Phase II Study of Intra-arterial Chemotherapy with Cisplatin and Mitomycin-C in Patients With Hepatocellular Carcinoma | HCC | [NCT00183885](https://clinicaltrials.gov/show/NCT00183885) |
| Sorafenib Plus Hepatic Arterial Infusion Versus Sorafenib for HCC With Major Portal Vein Tumor Thrombosis | HCC | [NCT03009461](https://clinicaltrials.gov/show/NCT03009461) |
| Effect of Surefire Infusion Device on Tumor Response to Regional Intra-arterial Therapy for Primary Liver Malignancies | HCC | [NCT02853500](https://clinicaltrials.gov/show/NCT02853500) |
| Study of Intra-Arterial Oxaliplatin Plus Capecitabine to Treat Liver Metastases from Colorectal Cancer | HCC | [NCT04701281](https://clinicaltrials.gov/show/NCT04701281) |
| Treatment With Hepatic Arterial Infusion of Oxaliplatin in Combination with Systemic FOLFIRI Chemotherapy and Bevacuzimab in Patients with Liver-only Colorectal Liver Metastases (CRLM): Conversion to Complete Resection in Patients with Initially Inoperable Liver-only CRLM. | HCC | [NCT04003792](https://clinicaltrials.gov/show/NCT04003792) |
| Chemotherapy or Not, Following Complete Treatment of Hepatic Cancer in Cirrhotic Patients | HCC | [NCT00470340](https://clinicaltrials.gov/show/NCT00470340) |
| Treatment of Cirrhosis-related Hepatocellular Carcinoma with the Intrahepatic Arterial Injection of an Emulsion of Lipiodol and Idarubicin: Phase I Study | HCC | [NCT02028949](https://clinicaltrials.gov/show/NCT02028949) |
| HAIC With Oxaliplatin, 5-FU and Bevacizumab Plus Intravenous Toripalimab for Advanced BTC | Hepatobiliary | [NCT04217954](https://clinicaltrials.gov/show/NCT04217954) |
| Intravenous or Hepatic Arterial Infusion of Fotemustine in Treating Patients with Unresectable Liver Metastases From Eye Melanoma | Melanoma | [NCT00110123](https://clinicaltrials.gov/show/NCT00110123) |
| Isolated Thoracic Perfusion (ITP-F) for MPM | Mesothelioma | [NCT02467426](https://clinicaltrials.gov/show/NCT02467426) |
| Transcatheter Intra-arterial Limb Infusion of Cisplatin for Extremity Osteosarcoma | Osteosarcoma | [NCT03909776](https://clinicaltrials.gov/show/NCT03909776) |
| Doxorubicin With Cisplatin, High-Dose Methotrexate, and Additional Risk-Adapted Outpatient Chemotherapy | Osteosarcoma | [NCT00673179](https://clinicaltrials.gov/show/NCT00673179) |
| Intra-arterial Versus Intravenous Chemotherapy for Locally Advanced Pancreatic Cancer | Pancreatic | [NCT02635971](https://clinicaltrials.gov/show/NCT02635971) |
| Intra-arterial Gemcitabine vs. IV Gemcitabine and Nab-Paclitaxel Following Radiotherapy for LAPC | Pancreatic | [NCT03257033](https://clinicaltrials.gov/show/NCT03257033) |
| Application of Intrahepatic Arterial Infusion Chemotherapy for Patients with High Risk of Liver Metastases After Pancreatic Cancer Surgery | Pancreatic | [NCT03687853](https://clinicaltrials.gov/show/NCT03687853) |
| Regional Versus Systemic Chemotherapy in the Treatment of Unresectable Pancreatic Cancer | Pancreatic | [NCT01665625](https://clinicaltrials.gov/show/NCT01665625) |
| Low-dose Intra-arterial Bevacizumab for Edema and Radiation Necrosis Therapeutic Intervention (LIBERTI) | Radiation Necrosis | [NCT02819479](https://clinicaltrials.gov/show/NCT02819479) |
| A Study of Sorafenib in Patients with Chemo-naive Metastatic Uveal Melanoma | Uveal Melanoma | [NCT01377025](https://clinicaltrials.gov/show/NCT01377025) |
